# External validation of the HACOR score and ROX index for predicting treatment failure in patients with coronavirus disease 2019 pneumonia managed on high-flow nasal cannula therapy: a multicenter retrospective observational study in Japan

**DOI:** 10.1186/s40560-024-00720-8

**Published:** 2024-02-15

**Authors:** Hiromu Okano, Ryohei Yamamoto, Yudai Iwasaki, Daisuke Irimada, Daisuke Konno, Taku Tanaka, Takatoshi Oishi, Hiroki Nawa, Akihiko Yano, Hiroaki Taniguchi, Masayuki Otawara, Ayaka Matsuoka, Masanori Yamauchi

**Affiliations:** 1 Department of Critical Care Medicine, St. Luke’s International Hospital, 9-1 Akashi-Cho, Chuo-Ku, Tokyo, 104-8560 Japan; 2 Department of Social Medical Sciences, Graduate School of Medicine, International University of Health and Welfare, 4-1-26 Akasaka, Minato-Ku, Tokyo 107-8402 Japan; 3 Center for Innovative Research for Communities and Clinical Excellence (CIRC2LE), Fukushima Medical University, Fukushima, 960-1295 Japan; 4 Department of Anesthesiology and Perioperative Medicine, Tohoku University Graduate School of Medicine, 1-1 Seiryo-Machi, Aoba-Ku, Sendai City, Miyagi 980-8574 Japan; 5 Department of Emergency and Critical Care Medicine, Nagoya University Graduate School of Medicine, Tsurumai-Cho 65, Syowa-Ku, Nagoya City, Aichi, 466-8550 Japan; 6 Department of Emergency and Critical Care Medicine, Jichi Medical University Saitmta Medical Center, 1-847, Amanuma-Cho, Oomiya-Ku, Saitama City, Saitama, 330-8503 Japan; 7 Department of Intensive Care Medicine, Kameda Medical Center, 929 Higashi-Cho, Kamogawa, Chiba 296-8602 Japan; 8 Department of General Medicine, Kochi Health Sciences Center, 2125-1 Ike, Kochi City, Kochi, 781-8555 Japan; 9 Department of Traumatology and Critical Care Medicine, National Defense Medical College Hospital, Namiki 3-2, Tokorozawa City, Saitama, 359-8513 Japan; 10 Emergency and Critical Care Center, Tokyo Metropolitan Hiroo Hospital, 2-34-10 Ebisu, Shibuya-Ku, Tokyo, 150-0013 Japan; 11 Department of Emergency and Critical Care Medicine, Faculty of Medicine, Saga University, Saga City, Saga 849-8501 Japan

**Keywords:** Coronavirus disease 2019, HACOR score, High-flow nasal cannula, ROX index, Tracheal intubation

## Abstract

**Background:**

The HACOR score for predicting treatment failure includes vital signs and acid–base balance factors, whereas the ROX index only considers the respiratory rate, oxygen saturation, and fraction of inspired oxygen (FiO_2_). We aimed to externally validate the HACOR score and ROX index for predicting treatment failure in patients with coronavirus disease 2019 (COVID-19) on high-flow nasal cannula (HFNC) therapy in Japan.

**Methods:**

This retrospective, observational, multicenter study included patients, aged ≥ 18 years, diagnosed with COVID-19 and treated with HFNC therapy between January 16, 2020, and March 31, 2022. The HACOR score and ROX index were calculated at 2, 6, 12, 24, and 48 h after stating HFNC therapy. The primary outcome was treatment failure (requirement for intubation or occurrence of death within 7 days). We calculated the area under the receiver operating characteristic curve (AUROC) and assessed the diagnostic performance of these indicators. The 2-h time-point prediction was considered the primary analysis and that of other time-points as the secondary analysis. We also assessed 2-h time-point sensitivity and specificity using previously reported cutoff values (HACOR score > 5, ROX index < 2.85).

**Results:**

We analyzed 300 patients from 9 institutions (median age, 60 years; median SpO_2_/FiO_2_ ratio at the start of HFNC therapy, 121). Within 7 days of HFNC therapy, treatment failure occurred in 127 (42%) patients. The HACOR score and ROX index at the 2-h time-point exhibited AUROC discrimination values of 0.63 and 0.57 (*P* = 0.24), respectively. These values varied with temporal changes—0.58 and 0.62 at 6 h, 0.70 and 0.68 at 12 h, 0.68 and 0.69 at 24 h, and 0.75 and 0.75 at 48 h, respectively. The 2-h time-point sensitivity and specificity were 18% and 91% for the HACOR score, respectively, and 3% and 100% for the ROX index, respectively. Visual calibration assessment revealed well calibrated HACOR score, but not ROX index.

**Conclusions:**

In COVID-19 patients receiving HFNC therapy in Japan, the predictive performance of the HACOR score and ROX index at the 2-h time-point may be inadequate. Furthermore, clinicians should be mindful of time-point scores owing to the variation of the models’ predictive performance with the time-point.

*Trial registration* UMIN (registration number: UMIN000050024, January 13, 2023)

**Supplementary Information:**

The online version contains supplementary material available at 10.1186/s40560-024-00720-8.

## Background

Patients diagnosed with severe coronavirus disease 2019 (COVID-19) pneumonia sometimes require intensive care unit (ICU) admission [1]. In such patients, a high-flow nasal cannula (HFNC) has often been needed before intubation [2]. Failure of HFNC therapy may result in delayed intubation and increased mortality [2]. Therefore, an early prediction of HFNC failure and determination of the appropriate timing of endotracheal intubation are important strategies for patient management.

The ROX index has been validated and widely used as a predictor of treatment failure (intubation) in patients with COVID-19 pneumonia treated with an HFNC [3]. This score is calculated using only three variables: respiratory rate, oxygen saturation (SpO_2_), and fraction of inspired oxygen (FiO_2_) [4, 5]. Other critical indicators, such as the level of consciousness, blood pressure, and acid–base balance, are also important parameters in determining the need for intubation. The HACOR score, a tool for predicting failure of noninvasive ventilation (NIV) and HFNC therapy, incorporates these additional factors [6, 7]. Previous studies have reported a high discriminatory performance of the HACOR score in patients with heart failure and acute respiratory failure [6, 7]. However, only one single center study by Valencia et al. [8] has externally validated the HACOR score in patients with COVID-19. Since the study was conducted at a single center, its generalizability was limited. Moreover, calibration assessment was not performed. Additionally, the decision-making process for treatment strategies during a pandemic is influenced by the medical setting, including the availability of limited resources such as ICU beds, mechanical ventilators, and healthcare workers [9, 10].

To enhance generalizability and transportability, we conducted a multicenter study to externally validate the HACOR score and ROX index for predicting treatment failure in patients with COVID-19 managed with HFNC therapy in Japan.

## Methods

### Design and setting

This retrospective, observational, multicenter study was conducted according to the Transparent Reporting of a Multivariate Prediction Model for Individual Prognosis or Diagnosis (TRIPOD) guidelines for prediction model validation [11]. The Institutional Review Board of Tohoku University (2022-I-265) and that of each center reviewed and approved the study.

The study was registered in the University Hospital Medical Information Network Clinical Trials Registry (UMIN-CTR ID UMIN000050024). The requirement for informed consent from all participants enrolled in this study was waived by the ethics committee owing to the retrospective study design.

### External validation cohort

We performed an external validation of two clinical prediction models, HACOR score and ROX index, using a multicenter, retrospective cohort study involving nine tertiary hospitals in Japan. These hospitals were the Yokohama Medical Center in Kanagawa, Tohoku University Hospital in Miyagi, National Defense Medical College Hospital in Saitama, Tokyo Metropolitan Hiroo Hospital in Tokyo, Saga University Hospital in Saga, Kochi Health Sciences Center in Kochi, Nagoya University Hospital in Aichi, Kameda Medical Center in Chiba, and Jichi Medical University Saitama Medical Center in Saitama. Additional information regarding the characteristics of these institutions can be found in Additional file 1: S1. The data were collected from January 16, 2020, to March 31, 2022, and during this period, the circulating strains of severe acute respiratory syndrome coronavirus 2 (SARS-CoV-2) in Japan were as follows: wild-type (January 2020 to February 2021); B.1.1.7 (alpha, March to April 2021): B.1.617.2 (delta, July to December 2021); and B.1.1.529 (omicron, January to June 2022) [12, 13].

### Study population

We included patients aged ≥ 18 years, who were diagnosed with COVID-19 and treated with an HFNC for > 2 h. The inclusion criteria were as follows: (1) patients aged ≥ 18 years; (2) confirmed SARS-CoV-2 infection detected using the real-time reverse transcription-polymerase chain reaction-loop-mediated isothermal amplification method or antigen test; and (3) treatment with an HFNC for at least 2 h. Patients who met any of the following conditions were excluded: (1) received NIV before HFNC therapy, (2) had do-not-intubate orders, and (3) were already extubated.

### Data collection

We utilized baseline data collected immediately before initiating HFNC therapy, which included patient characteristics such as age, sex, height, weight, comorbidities, and vital signs. Blood sampling data, Sequential Organ Failure Assessment (SOFA) scores, and Acute Physiology and Chronic Health Evaluation (APACHE) II scores were collected within the first 24 h before initiating HFNC therapy. Furthermore, we collected the data including those on vital signs, arterial blood gas, and oxygen device settings at multiple time-points after the initiation of HFNC therapy, specifically at 2, 6, 12, 24, and 48 h.

### Outcome measurement

The outcome, treatment failure, was defined as either intubation or death within 7 days. In a previous study [8], treatment failure was defined as HFNC therapy failure, including the need for mechanical ventilation and death within 7 days [14]. According to the study design, the decision regarding intubation was at the discretion of the clinicians at each participating site.

### Predictor variables

The HACOR score and ROX index were calculated at the 2, 6, 12, 24, and 48-h time-points following the commencement of HFNC therapy. The scores were measured for each of these time periods until the withdrawal of HFNC.

### Statistical analyses

Baseline characteristics are presented as descriptive statistics including the median (interquartile range) for continuous variables and frequency (percentage) for categorical variables.

### Sample size

According to the TRIPOD guidelines [11], we defined the research period from January 16, 2020, to March 31, 2022. For external validation, we determined a study sample size that included a minimum of 100 patients with the outcome of interest and 100 without the outcome. All patients were recruited during the specified period with the objective of collecting data from more than 200 patients. Owing to the uncertainty regarding the required sample size required for each facility to achieve this target, we extended the maximum period to the time when COVID-19 was first confirmed in Japan.

### Validation of the models

We evaluated the performance of the HACOR score and ROX index at each time-point. To avoid multiple testing, we predetermined using that the primary analysis would focus on the prediction at the 2-h time point, while predictions at other time-points would be considered the secondary analyses. The 2-h time point prediction was chosen as the primary analysis because of the clinical importance of early prediction and its use in existing studies [7, 8]. To estimate the discrimination ability between patients with and without treatment failure, we calculated the area under the receiver operating characteristic curve (AUROC). We interpreted AUROC values greater than 0.9 as high, 0.7–0.9 as moderate, 0.5–0.7 as low, and 0.5 as a chance result [15]. Additionally, we evaluated the predictive performance, including sensitivity, specificity, and positive and negative predicted values, utilizing the threshold criteria established in previous studies for the HACOR score versus those for the ROX index as follows: > 5 versus < 2.85 at 2 h; > 5 versus < 3.47 at 6 h; and > 5 versus < 3.85 at 12 h, respectively [4, 7]. Because of limited evidence regarding the cutoff points at 24 and 48 h, we employed the threshold established at 12 h. To evaluate the calibration, we compared the observed proportion of treatment failures with predicted risks. Calibration graphs were visually assessed and considered to be well calibrated if the observed treatment failures increased as the risk of the group increased. In clinical practice, the HACOR score is utilized as a point-based scoring system, whereas the ROX index is not assigned specific numerical values but is instead assessed using a three-tiered risk categorization [16]. Consequently, the calculated values are employed for assessing the HACOR score, whereas the ROX index is evaluated based on its categorization. For the HACOR score, patients were stratified according to individual score groups. Concerning the ROX index, we categorized patients into three risk groups based on their ROX index as follows: high (ROX index < 3.85), intermediate (3.85 ≤ ROX index ≤ 4.88), and low (ROX index > 4.88) [4, 16].

### Comparison of the two models

The HACOR score and ROX index were compared by comparing the AUROC using the Delong method [17].

### Sensitivity analysis

We conducted a sensitivity analysis to ensure the robustness of the results of the primary and secondary outcome analyses. Given the subjective nature of the decision to intubate, we defined clinically important intubation as having met at least one of the following criteria: loss or impaired level of consciousness (a Glasgow Coma Scale score of < 8), hypotension (a systolic arterial blood pressure of < 90 mmHg or a mean arterial blood pressure of < 65 mmHg), a respiratory rate of > 40 /min, or hypoxia with an SpO_2_ of < 92% despite an FiO_2_ of 1.0 or a pH < 7.35, by blood gas analysis [6]. We performed the same analysis using clinically significant intubation or death within 7 days as the outcome. As part of a sensitivity analysis, we conducted the same analysis with intubation as the outcome. In addition, a sensitivity analysis was performed excluding facilities that used the ROX index as a treatment guide. Multiple imputations were performed for missing values using multiple imputations by chained equation with 50 iterations that generated 100 datasets for imputed missing values [18, 19]. Analyses were performed using R software, version 4.1.1 (The R Foundation for Statistical Computing, Vienna, Austria, https://www.R-project.org/).

## Results

### Patient characteristics

**Fig. 1:**
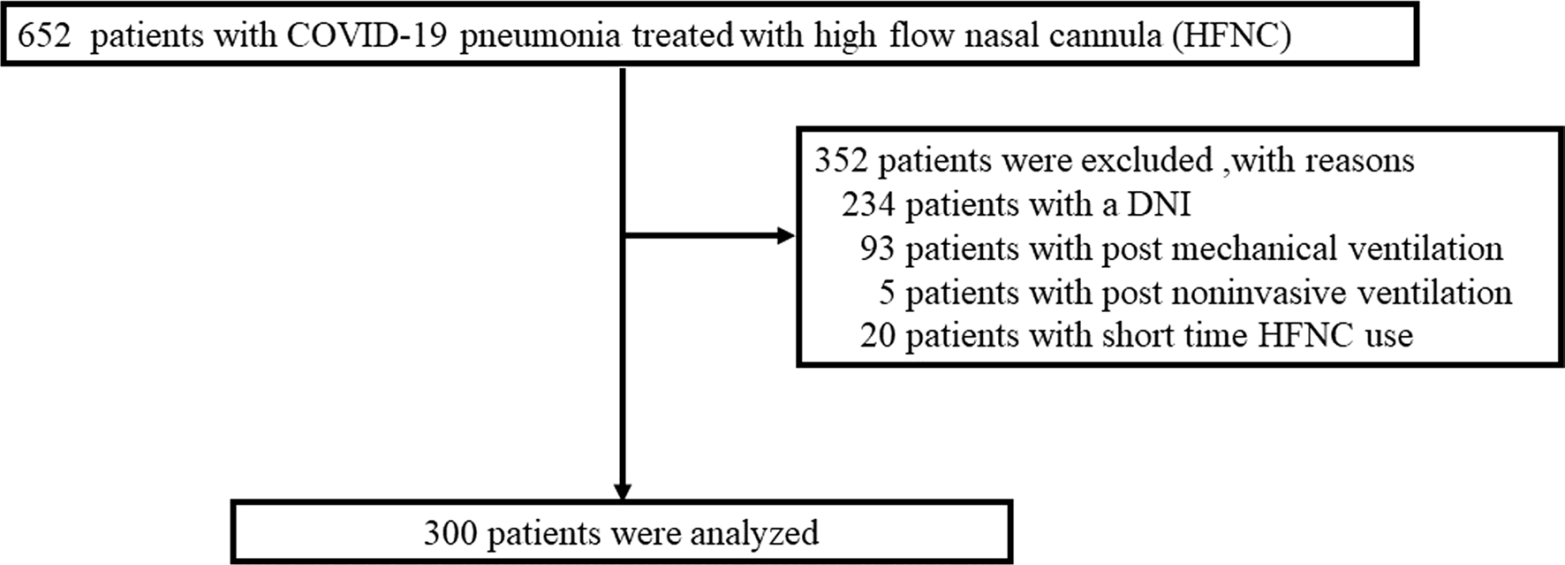
Flow diagram of the study participant selection process. To achieve this enrollment, we set the research period from January 16, 2020, to March 31, 2022. Upon collecting cases during this period, the actual enrollment reached 300 participants. DNI: do-not-intubate

During the study period, 652 patients received HFNC therapy. Of these, 352 were excluded; ultimately, the data of 300 patients were analyzed (Fig. 1). Seventy-six percent (228/300) patients were male, and the median age was 60 (IQR, 51–70) years. The median number of days from onset to hospitalization was 7 (IQR 5–10) days. Notably, vaccines against COVID-19 were not widely available in Japan for more than half of the study period (vaccination against COVID-19 began on February 17, 2021), and 89% (245/276) of the patients were unvaccinated (Table 1). While the HACOR score did not affect treatment decisions at any of the facilities, the ROX index influenced treatment policies at two participating sites (Additional file 1: S1).

**Table 1:** Baseline characteristics of patients with COVID-19 pneumonia treated with high-flow nasal cannula

Characteristic	*N*	Overall, *N* = 300	Non-failure, *N* = 173	Failure, *N* = 127
Age, years	300	60 (51, 70)	60 (51, 69)	61 (50, 72)
Male	300	228 (76)	130 (75)	98 (77)
Body mass index, kg/m^2^	286	25.2 (22.6, 29.3)	25.0 (22.6, 28.6)	25.7 (22.8, 31.7)
Hypertension	300	146 (49)	83 (48)	63 (50)
Chronic pulmonary disease	300	28 (9.3)	11 (6.4)	17 (13)
Any tumor	300	20 (6.7)	8 (4.6)	12 (9.4)
Chronic heart failure	300	13 (4.3)	9 (5.2)	4 (3.1)
Diabetes	300	75 (25)	42 (24)	33 (26)
Dementia	300	9 (3.0)	7 (4.0)	2 (1.6)
Delirium	299	9 (3.0)	5 (2.9)	4 (3.2)
Vaccination, doses	276			
0		245 (89)	138 (87)	107 (91)
1		6 (2.2)	4 (2.5)	2 (1.7)
2		23 (8.3)	16 (10)	7 (6.0)
3		2 (0.7)	1 (0.6)	1 (0.9)
SOFA score	282	2 (2, 3)	2 (2, 3)	2.00 (2.00, 3.00)
APACHE2	254	9 (6, 13)	9 (6, 13)	8 (6, 12)
Heart rate, bpm/min	284	82 (72, 92)	80 (71, 91)	84 (78, 99)
Glasgow come scale	291	15 (15, 15)	15(15, 15)	15 (15, 15)
Body temperature, °C	282	37.0 (36.6, 37.8)	36.8 (36.5, 37.6)	37.2 (36.7, 38.0)
Respiratory rate, bpm/min	278	25 (21, 30)	25 (20, 30)	25 (22, 30)
SpO_2_	295	92 (90., 95.)	93 (91, 96)	91 (88, 93)
FiO_2_	280	0.75 (0.50, 0.95)	0.60 (0.44, 0.95)	0.90 (0.60, 0.95)
SpO_2_/FiO_2_	280	121 (96, 184)	145 (100, 211)	101 (93, 158)
Number of days from onset to hospitalization	300	7 (5, 10)	8 (4, 10)	6 (5, 8)
Number of days from admission to HFNC	300	0.83 (0.62, 1.65)	0.83 (0.65, 1.56)	0.84 (0.60, 1.78)
Total bilirubin, mg/dL	295	0.56 (0.43, 0.70)	0.57 (0.49, 0.72)	0.51 (0.40, 0.70)
Blood urea nitrogen, mg/dL	295	19 (14, 26)	19 (13, 24)	21 (15, 28)
Creatinine, mg/dL	294	0.81 (0.66, 1.07)	0.78 (0.63, 0.95)	0.85 (0.70, 1.15)
Platelets, 10^3^/µL	295	169 (108, 226)	179 (89, 246)	160 (116, 220)

Data are shown as n (%) or median (interquartile range). APACHE: Acute Physiology and Chronic Health Evaluation; FiO_2_: fraction of inspired oxygen; SOFA: Sequential Organ Failure Assessment; SpO_2_: oxygen saturation

### Treatment failure

The time to intubation was 27 (IQR 13–37) h, and 42% (127/300) of the patients were intubated by day 7. The 7-day and in-hospital mortality were 2% (6/300) and 14% (43/300), respectively. A total of 42% (127/300) patients experienced treatment failure (Table 2), and 45% (136/300) patients either required endotracheal intubation or died within 28 days (Table 2).

**Table 2:** Intubation, length of stay and mortality associated with COVID-19 patients with high-flow nasal cannula

Clinical course	Number	Overall *n* = 300
Time to intubation, hour	136*	27 (13 to 72)
Intubated within day 7	300	127 (42)
Intubated within day 28	300	133 (44)
Hospital length of stay, day	300	17 (11 to 30)
7 day mortality	300	6 (2.0)
28 day mortality	300	25 (8.3)
In-hospital mortality	300	43 (14)
Treatment failure within day 7	300	127 (42)
Treatment failure within day 28	300	136 (45)

Data are shown as *n* (%) or median (interquartile range)

Treatment failure was defined as either intubation or death within 7 days

*Calculated only for intubated patients

### Predictive performance of the HACOR score and ROX index

The HACOR score and ROX index values at each time point are presented in Additional file 1: S2. In primary analysis, the discrimination of the HACOR score and ROX index at the 2-h time-point was indicated by AUROC values of 0.63 and 0.57 (*P* = 0.24), respectively. In the secondary analyses, the temporal changes in the discrimination of the HACOR score and the ROX index were 0.58 and 0.62 at 6 h (*P* = 0.045), 0.70 and 0.68 at 12 h (*P* = 0.37), 0.68 and 0.69 at 24 h (*P* = 0.63), and 0.75 and 0.75 at 48 h (*P* = 0.84), respectively (Table 3). The sensitivity and specificity at 2 h were 18% and 91% for the HACOR score and 3.9% and 100% for the ROX index, respectively (Table 4).

**Table 3:** The area under the receiver operating characteristic curve of each predictions

Time point	HACOR score AUROC (95% CI)	ROX index AUROC (95% CI)	*P*-value* (HACOR versus ROX)
2 h	0.63 (0.54 to 0.71)	0.57 (0.51 to 0.64)	0.24
6 h	0.58 (0.49 to 0.68)	0.62 (0.55 to 0.69)	0.045
12 h	0.70 (0.62 to 0.78)	0.68 (0.62 to 0.75)	0.37
24 h	0.68 (0.58 to 0.78)	0.69 (0.61 to 0.77)	0.63
48 h	0.75 (0.64 to 0.86)	0.75 (0.65 to 0.85)	0.84

AUROC: the area under the receiver operating characteristic curve; CI: confidence interval

*Delong test

**Table 4:** Other discriminations of the HACOR score and ROX index

Time point	HACOR score				ROX index			
	Sensitivity	Specificity	PPV	NPV	Sensitivity	Specificity	PPV	NPV
2 h	0.18	0.91	0.61	0.60	0.039	1	1	0.59
6 h	0.12	0.96	0.63	0.64	0.058	0.99	0.86	0.64
12 h	0.16	0.94	0.6	0.67	0.022	0.99	0.67	0.65
24 h	0.22	0.93	0.54	0.74	0.077	1	1	0.73
48 h	0.34	0.95	0.65	0.83	0.16	0.99	0.86	0.80

NPV: negative predictive value; PPV: positive predictive value

we evaluated the predictive performance, including sensitivity, specificity, and positive and negative predicted values, utilizing the threshold criteria, which the HACOR score versus those for the ROX index as follows: > 5 versus < 2.85 at 2 h; > 5 versus < 3.47 at 6 h; and > 5 versus < 3.85 at 12 h, respectively. Because of limited evidence regarding the cutoff points at 24 and 48 h, we employed the threshold established at 12 h

**Fig. 2:**
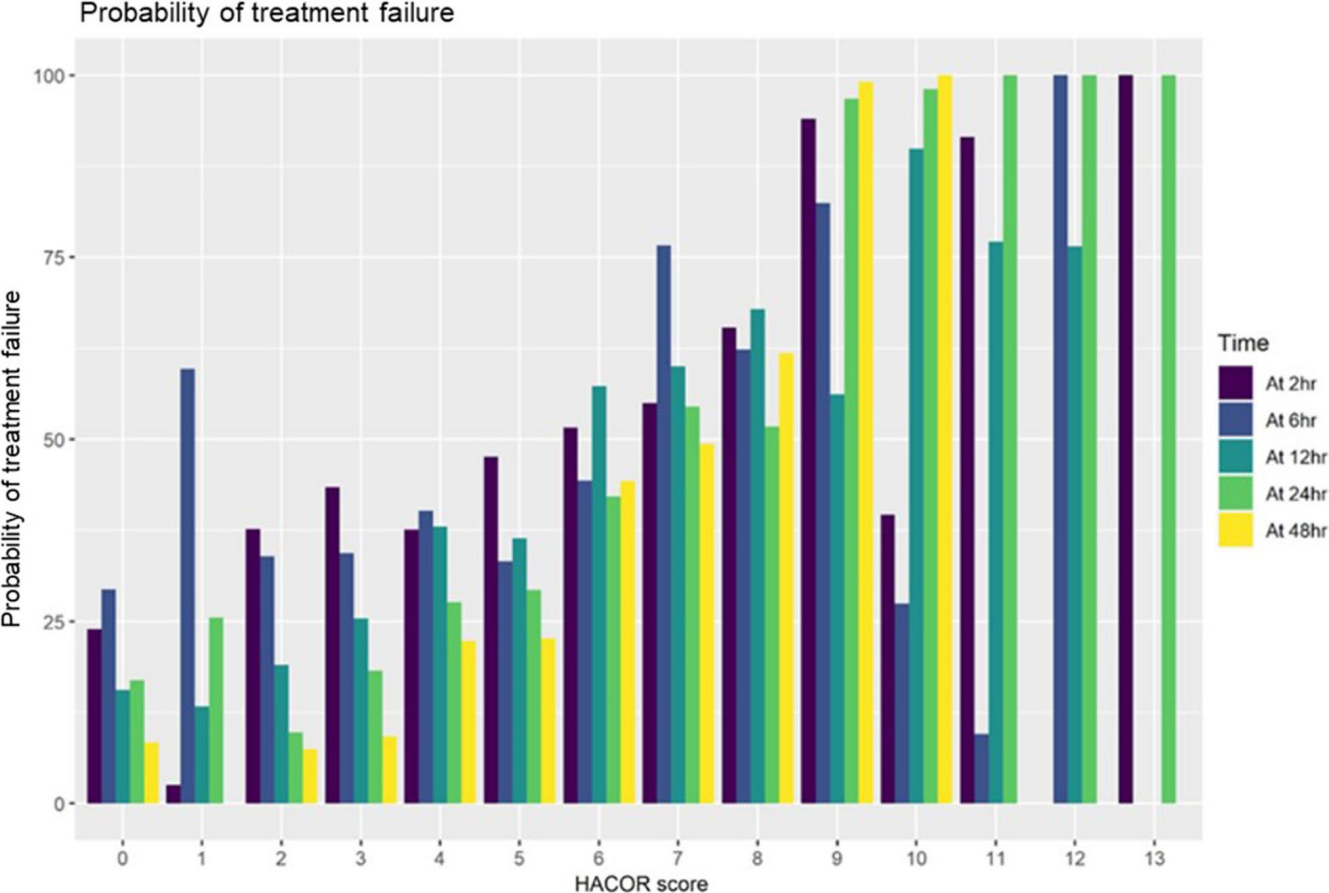
Calibration of the HACOR score. Calibration of the HACOR score is shown; 27% of patients experienced treatment failure even when the HACOR score was 0 at 2 h. An increasing probability in treatment failure was observed with increasing scores. Treatment failure was defined as either intubation or death within 7 days

**Fig. 3:**
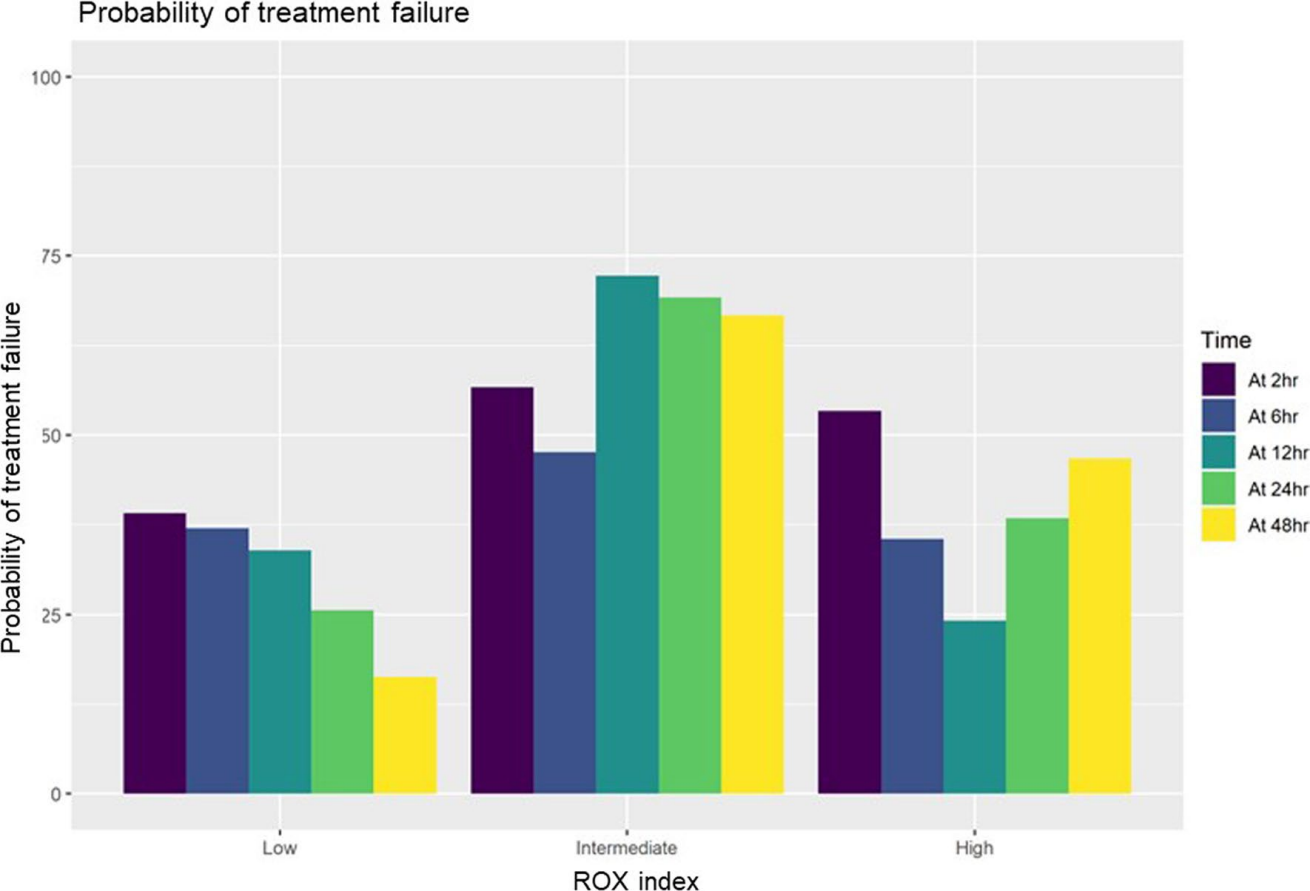
Calibration of the ROX index. We categorized patients into three risk groups based on their ROX index: high (ROX index < 3.85), intermediate (3.85 ≤ ROX index ≤ 4.88), and low (ROX index > 4.88). Calibration of the ROX index is illustrated; at 2 h, approximately 40%, 57%, and 53% of patients in the low-, intermediate-, and high-risk groups, respectively, experienced treatment failure, indicating that the ROX index was not well calibrated. Treatment failure was defined as either intubation or death within 7 days

Calibration of the HACOR score is shown in Fig. 2. The figure displays an observable trend of increasing intubation rate with an increasing score, although 24% of the patients required intubation even when the HACOR score was 0 at 2 h. By contrast, Fig. 3 illustrates that the ROX index was not well calibrated because approximately 40%, 57%, and 53% of the patients in the low-, intermediate-, and high-risk groups, respectively, experienced treatment failure in 2 h.

### Sensitivity analyses

Of the 42% (127/300) patients who were intubated within 7 days, 80 met the criteria for clinically significant intubation (Additional file 1: S3). When the outcome was clinically important intubation or death within 7 days, the discrimination of the HACOR score and ROX index at the 2-h time-point, the primary outcome, was indicated by AUROC values of 0.65 and 0.63, respectively (*P* = 0.24). The temporal changes in the discrimination of the HACOR score and ROX index, the secondary outcome, were 0.57 and 0.65 at 6 h (*P* = 0.045), 0.69 and 0.73 at 12 h (*P* = 0.37), 0.67 and 0.74 at 24 h (*P* = 0.63), and 0.73 and 0.76 at 48 h (*P* = 0.84), respectively (Additional file 1: S4). I In a sensitivity analysis using intubation as an outcome, as no deaths occurred within day 7, treatment failure within day 7 and intubation within day 7 had the same results (Tables 3 and 4). The sensitivity and specificity at 2 h were 18% and 91% for the HACOR score and 3.9% and 100% for the ROX index, respectively (Table 4). The sensitivity analysis results excluding facilities that used the ROX index as a treatment guide were in Additional file 1: S5 and S6, showing AUROCs at 2 h of 0.64 for the HACOR score and 0.61 for the ROX index.

## Discussion

### Summary of the key findings

Our study showed that in Japanese patients with COVID-19 on HFNC therapy, the HACOR score and ROX index had low discrimination and poor calibration at 2 h; however, their AUROC tended to increase over time. The predictive performance of the HACOR score and ROX index in Japanese patients with COVID-19 on HFNC therapy may be inadequate due to low discrimination and poor calibration. The novelty of our study is that in addition to the discriminative ability of the HACOR score and ROX index, we also evaluated their calibration ability. At 2 h, even among patients with an HACOR score of 0, approximately 25% were intubated, and in evaluating the ROX index, even in the low-risk patient group, approximately 40% were intubated. Calibration of the HACOR score may improve over time; however, no improvement was observed in the ROX index.

### Discussion of the results in relation to previous findings

A previous study [20] has suggested the utility of the ROX index in predicting the failure of HFNC therapy in patients with COVID-19. According to a prior systematic review [3] that included eight cohort studies with 1301 patients, the ROX index showed moderate ability to discriminate between outcomes, with a summary AUROC of 0.81. However, the ability of the HACOR score to predict failure of HFNC therapy in patients with COVID-19 receiving HFNC has only been investigated in one study [8].

Valencia et al. [8] reported the discriminatory ability of the HACOR score for treatment failure after 2 h of HFNC therapy in patients with COVID-19. In their study, the HACOR score demonstrated an AUROC of 0.71. Our data’s poorer discriminatory ability could be explained by several reasons. First, there were differences in the intubation criteria, which are a part of the treatment failure criteria. Valencia et al. [8] evaluated the HACOR score and ROX index after 2 h of HFNC therapy, and if there was no improvement in the signs of muscle fatigue, SaO_2_ (> 90%), and PaO_2_/FiO_2_, endotracheal intubation was performed. The criteria used to determine intubation were similar to those for the HACOR score and the ROX index. When the index test and outcome were closely related, the AUROC tended to be higher. In our study, owing to the nature of the study design, there were no standardized intubation criteria. This may have influenced the lower discrimination power. In the sensitivity analysis, an improvement in the AUROC was observed when clinically important intubation criteria were used. Second, there is a possibility of diagnostic review bias due to the lack of blinding during the evaluation [21]. In prediction model studies, outcomes should ideally be assessed in a blinded manner, without prior knowledge of the predictors [22]. This approach prevents the predictors from influencing the outcome assessment, thereby preventing biased estimation in the association between predictors and outcomes. In the study by Valencia et al. [8], physicians were aware of the patients' HACOR score and ROX index when deciding to intubate. In such a setting, there is a potential for these scores to influence the decision-making process, leading to an overestimation of reported accuracy estimates [21]. In our study, we performed a sensitivity analysis by excluding facilities that used the ROX index as a treatment indicator. The results showed a trend toward improved discrimination over time, similar to the main analysis.

The lack of significant differences in the predictive performance of the HACOR score and ROX index at 2-h time point could be attributed to the specific nature of COVID-19. In COVID-19, low oxygen levels without accompanying respiratory distress, increased breathing effort, or elevated respiratory rate are observed, a phenomenon known as “happy hypoxemia [23, 24]”. Moreover, in most of our patients, the median GCS was 15 and the heart rate did not exceed 120 bpm (Table 1). In such cases, the predictive performance of the HACOR score may be similar to that of the ROX index. Further research is required to evaluate the effectiveness of the HACOR score in patients with COVID-19.

The predictive performance of the ROX index improved over time. This finding is consistent with that of a previous study [20]. One possible explanation for the improved performance of the ROX index in later time windows is the initial undifferentiated state of patients. As HFNC therapy progresses, it allows the typical course of COVID-19 to unfold, offering an opportunity for specific treatments, such as steroids and antiviral drugs, to demonstrate their effectiveness [20]. Another contributing factor could be the inherent nature of predictive models; their accuracy typically improves when the predicted event is closer to the data’s time point. Essentially, more recent data tend to better reflect the current clinical scenario, leading to a more precise prediction.

A higher likelihood of treatment failure was observed in the intermediate-risk group. Although a previous study [25] indicated that prophylactic intubation does not improve patient outcomes, our findings suggest that early elective intubation may have been performed to protect healthcare workers from potential aerosol transmission of COVID-19 during HFNC therapy. This interpretation was supported by our sensitivity analysis, which revealed that 47 of 127 patients were intubated without meeting the criteria for clinically important intubation. On the other hand, the reason for the lower incidence of treatment failures in high-risk group as opposed to intermediate-risk group remains unclear. The decision for intubation during a pandemic may have taken into account not only the risk of the ROX index, but also other factors such as infection control, ventilator availability, facility policies, and manpower [9, 10]; however, data supporting these factors were not recorded in this study.

### Implications for clinical practice and future research

The incidence of treatment failure among patients initiating HFNC therapy was 42%, and a significant number of patients experienced treatment failure despite having a HACOR score of 0 or being classified as low-risk according to the ROX index. These groups still present risks that should be carefully managed, suggesting that the use of predictive models may not change clinical decision-making. For example, if a patient scores 0 on the HACOR scale, there remains a 24% chance that they might require intubation. Therefore, a score of 0 does not justify overlooking careful observation. Moreover, it may be inappropriate to use these models to rule out treatment failure. As the predictive performance of these models varied depending on the time-point, clinicians need to be mindful of the time-point score they are utilizing. An improved version of the HACOR score, the updated HACOR score [26], has been recently published, but has only been assessed in NIV. More accurate models need to be developed to predict HFNC treatment failure and to support clinical decision-making.

### Limitations

Our study had certain limitations. First, the data might exhibit heterogeneities. Especially, there was heterogeneity in the criteria for initiating HFNC therapy between facilities. However, external validation of predictive models must be performed in real-world settings. Second, the generalizability might be limited. Although we analyzed data from nine centers over two years, further validation over different time periods and geographical locations is essential. Third, the decision to perform intubation was subjective. Although sensitivity analysis demonstrated an improved AUROC for the ROX index, its predictive performance remained inadequate. The HFNC initiation settings were not standardized across the participating facilities, with HFNC flow settings varying from 30 to 60 L/min. Given that HFNC flow settings have been reported to play an important role in physiological effects, concerns remain regarding the possibility that differences in flow settings may have affected the results [27, 28]. However, the influence of flow settings could be minimal, as previous systematic reviews that did not account for HFNC flow settings have still demonstrated reasonable predictive accuracy [20]. Fourth, the study design was retrospective, resulting in missing data. We adhered to the recommended methods for handling missing data and conducted our analysis based on the TRIPOD guidelines [11]. Finally, in  Japan, vaccinations have been administered since July 2021; therefore, the study included data obtained before the availability of vaccines, potentially limiting the generalizability of our findings to the current era.

## Conclusions

In patients with COVID-19 managed on HFNC therapy in Japan, the predictive performance of the HACOR score and ROX index at 2 h may be inadequate.

Consequently, these models may not be reliable for excluding early treatment failure. However, the discrimination of both scores tended to improve over time. It is important for healthcare providers to consider the timing of score assessment, as the accuracy of these predictive models varied depending on when they were used.

### Supplementary Information

**Additional file 1. S1:** Characteristics of each institution and ICU. **S2: **Test values at each measurement point and HACOR score and ROX Index. **S3: **Clinical important intubation and treatment failure. **S4:** The area under the receiver operating characteristic curve of each predictions for the outcomes, which were clinically important intubation or death within 7 days. **S5:** The area under the receiver operating characteristic curve of each predictions for excluding facilities that used ROX index as a guide for treatment. **S6:** Discrimination of the HACOR score and ROX index predictions excluding facilities that used use ROX index as a guide for treatment.

## Data Availability

The datasets generated and/or analyzed during the current study are not publicly available, as they may be used for a post hoc analysis by the co-authors, but are available from the corresponding author upon reasonable request.

## Abbreviations

AUROC: Area under the receiver operating characteristic curve
APACHE: Acute Physiology and Chronic Health Evaluation
CI: Confidence interval
COVID-19: Coronavirus disease 2019
FiO_2_: Fraction of inspired oxygen
HFNC: High-flow nasal cannula
ICU: Intensive care unit
IQR: Interquartile range
NIV: Noninvasive positive pressure ventilation
NPV: Negative predictive value
PPV: Positive predictive value
SOFA: Sequential Organ Failure Assessment
SpO_2_: Oxygen saturation
