# Supplementary material for: External validation of the HACOR score and ROX index for predicting treatment failure in patients with coronavirus disease 2019 pneumonia managed on high-flow nasal cannula therapy: a multicenter retrospective observational study in Japan
Source: J Intensive Care. 2024 Feb 15;12:7. doi: 10.1186/s40560-024-00720-8 (PMC10870626; doi:10.1186/s40560-024-00720-8)
Supplement: Supplementary file 1 — Additional file 1. S1: Characteristics of each institution and ICU. S2: Test values at each measurement point and HACOR score and ROX Index. S3: Clinical important intubation and treatment failure. S4: The area under the receiver operating characteristic curve of each predictions for the outcomes, which were clinically important intubation or death within 7 days. S5: The area under the receiver operating characteristic curve of each predictions for excluding facilities that used ROX index as a guide for treatment. S6: Discrimination of the HACOR score and ROX index predictions excluding facilities that used use ROX index as a guide for treatment. [file 40560_2024_720_MOESM1_ESM.pptx]

## Slide 1
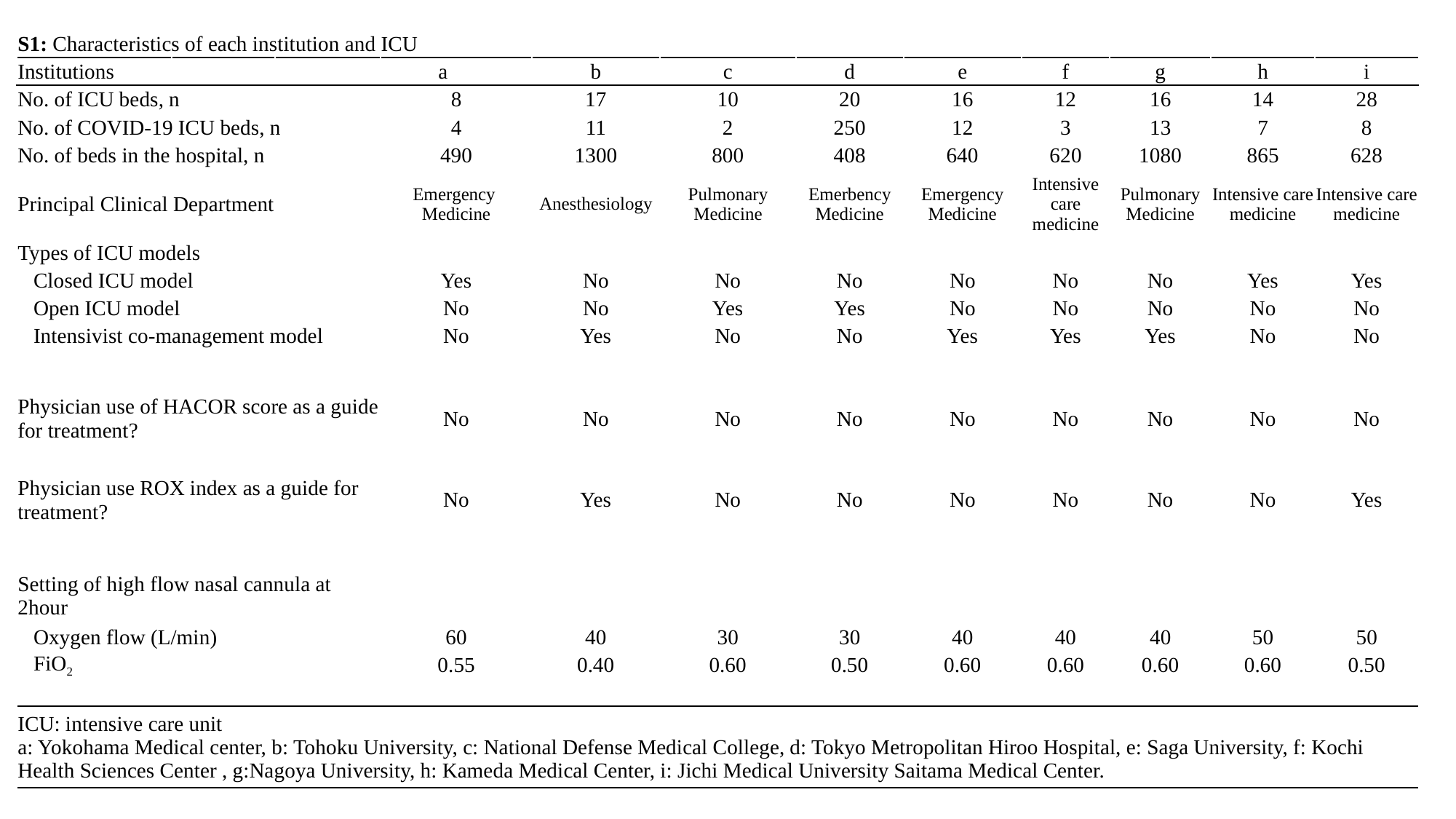

| S1: Characteristics of each institution and ICU | | | | | | | | | | | | | |
| --- | --- | --- | --- | --- | --- | --- | --- | --- | --- | --- | --- | --- | --- |
| Institutions | | | | | a | b | c | d | e | f | g | h | i |
| No. of ICU beds, n | | | | | 8 | 17 | 10 | 20 | 16 | 12 | 16 | 14 | 28 |
| No. of COVID-19 ICU beds, n | | | | | 4 | 11 | 2 | 250 | 12 | 3 | 13 | 7 | 8 |
| No. of beds in the hospital, n | | | | | 490 | 1300 | 800 | 408 | 640 | 620 | 1080 | 865 | 628 |
| Principal Clinical Department | | | | | Emergency Medicine | Anesthesiology | Pulmonary Medicine | Emerbency Medicine | Emergency Medicine | Intensive care medicine | Pulmonary Medicine | Intensive care medicine | Intensive care medicine |
| Types of ICU models | | | | | | | | | | | | | |
| Closed ICU model | | | | | Yes | No | No | No | No | No | No | Yes | Yes |
| Open ICU model | | | | | No | No | Yes | Yes | No | No | No | No | No |
| Intensivist co-management model | | | | | No | Yes | No | No | Yes | Yes | Yes | No | No |
| | | | | | | | | | | | | | |
| Physician use of HACOR score as a guide for treatment? | | | | | No | No | No | No | No | No | No | No | No |
| Physician use ROX index as a guide for treatment? | | | | | No | Yes | No | No | No | No | No | No | Yes |
| | | | | | | | | | | | | | |
| Setting of high flow nasal cannula at 2hour | | | | | | | | | | | | | |
| Oxygen flow (L/min) | | | | | 60 | 40 | 30 | 30 | 40 | 40 | 40 | 50 | 50 |
| FiO2 | | | | | 0.55 | 0.40 | 0.60 | 0.50 | 0.60 | 0.60 | 0.60 | 0.60 | 0.50 |
| | | | | | | | | | | | | | |
| ICU: intensive care unit a: Yokohama Medical center, b: Tohoku University, c: National Defense Medical College, d: Tokyo Metropolitan Hiroo Hospital, e: Saga University, f: Kochi Health Sciences Center , g:Nagoya University, h: Kameda Medical Center, i: Jichi Medical University Saitama Medical Center. | | | | | | | | | | | | | |

## Slide 2
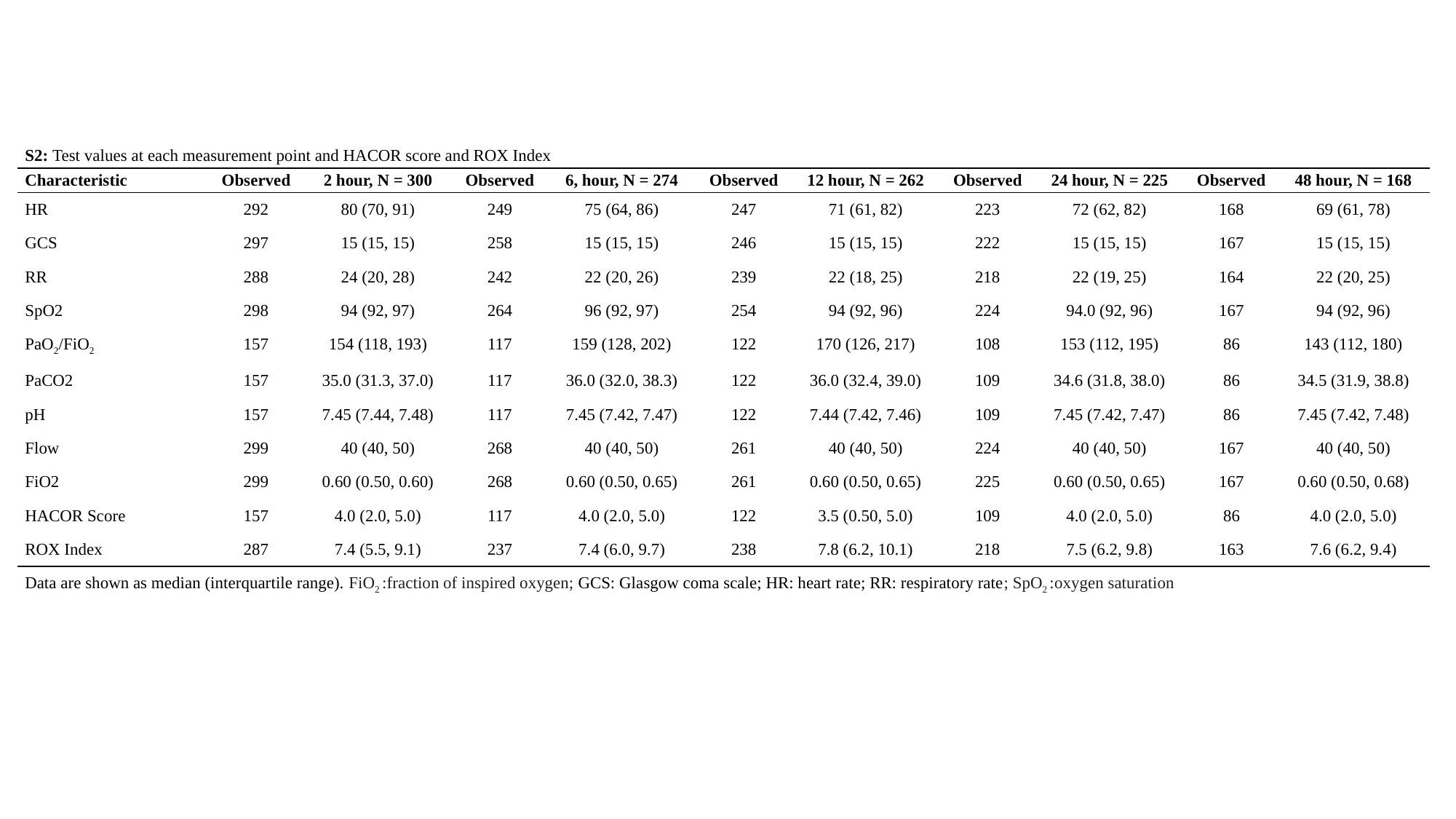

| S2: Test values at each measurement point and HACOR score and ROX Index | | Visit | Visit | Visit | Visit | Visit | Visit | Visit | Visit | Visit |
| --- | --- | --- | --- | --- | --- | --- | --- | --- | --- | --- |
| Characteristic | Observed | 2 hour, N = 300 | Observed | 6, hour, N = 274 | Observed | 12 hour, N = 262 | Observed | 24 hour, N = 225 | Observed | 48 hour, N = 168 |
| HR | 292 | 80 (70, 91) | 249 | 75 (64, 86) | 247 | 71 (61, 82) | 223 | 72 (62, 82) | 168 | 69 (61, 78) |
| GCS | 297 | 15 (15, 15) | 258 | 15 (15, 15) | 246 | 15 (15, 15) | 222 | 15 (15, 15) | 167 | 15 (15, 15) |
| RR | 288 | 24 (20, 28) | 242 | 22 (20, 26) | 239 | 22 (18, 25) | 218 | 22 (19, 25) | 164 | 22 (20, 25) |
| SpO2 | 298 | 94 (92, 97) | 264 | 96 (92, 97) | 254 | 94 (92, 96) | 224 | 94.0 (92, 96) | 167 | 94 (92, 96) |
| PaO2/FiO2 | 157 | 154 (118, 193) | 117 | 159 (128, 202) | 122 | 170 (126, 217) | 108 | 153 (112, 195) | 86 | 143 (112, 180) |
| PaCO2 | 157 | 35.0 (31.3, 37.0) | 117 | 36.0 (32.0, 38.3) | 122 | 36.0 (32.4, 39.0) | 109 | 34.6 (31.8, 38.0) | 86 | 34.5 (31.9, 38.8) |
| pH | 157 | 7.45 (7.44, 7.48) | 117 | 7.45 (7.42, 7.47) | 122 | 7.44 (7.42, 7.46) | 109 | 7.45 (7.42, 7.47) | 86 | 7.45 (7.42, 7.48) |
| Flow | 299 | 40 (40, 50) | 268 | 40 (40, 50) | 261 | 40 (40, 50) | 224 | 40 (40, 50) | 167 | 40 (40, 50) |
| FiO2 | 299 | 0.60 (0.50, 0.60) | 268 | 0.60 (0.50, 0.65) | 261 | 0.60 (0.50, 0.65) | 225 | 0.60 (0.50, 0.65) | 167 | 0.60 (0.50, 0.68) |
| HACOR Score | 157 | 4.0 (2.0, 5.0) | 117 | 4.0 (2.0, 5.0) | 122 | 3.5 (0.50, 5.0) | 109 | 4.0 (2.0, 5.0) | 86 | 4.0 (2.0, 5.0) |
| ROX Index | 287 | 7.4 (5.5, 9.1) | 237 | 7.4 (6.0, 9.7) | 238 | 7.8 (6.2, 10.1) | 218 | 7.5 (6.2, 9.8) | 163 | 7.6 (6.2, 9.4) |
| Data are shown as median (interquartile range). FiO2 :fraction of inspired oxygen; GCS: Glasgow coma scale; HR: heart rate; RR: respiratory rate; SpO2 :oxygen saturation | | | | | | | | | | |

## Slide 3
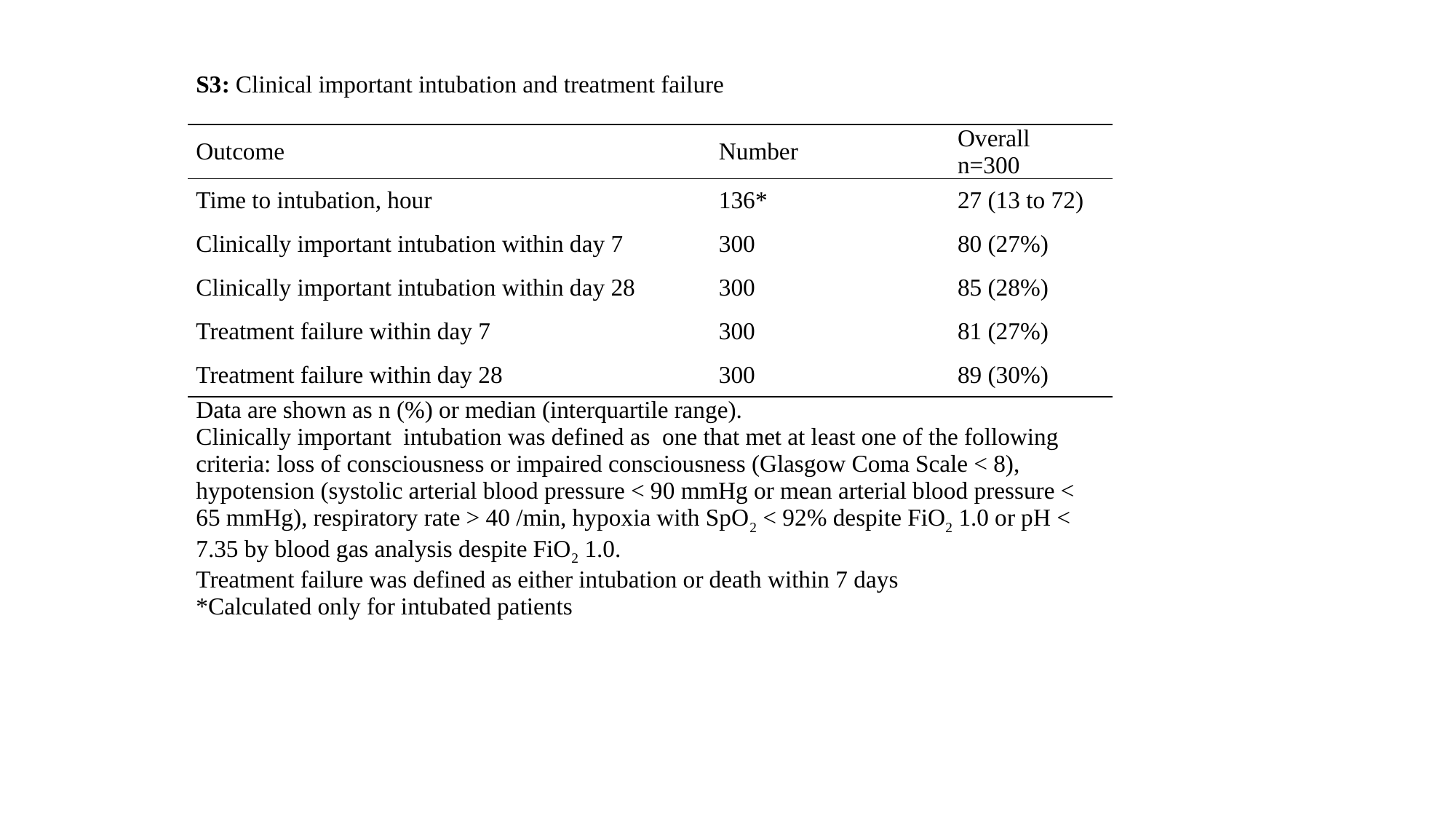

| S3: Clinical important intubation and treatment failure | | |
| --- | --- | --- |
| Outcome | Number | Overall n=300 |
| Time to intubation, hour | 136\* | 27 (13 to 72) |
| Clinically important intubation within day 7 | 300 | 80 (27%) |
| Clinically important intubation within day 28 | 300 | 85 (28%) |
| Treatment failure within day 7 | 300 | 81 (27%) |
| Treatment failure within day 28 | 300 | 89 (30%) |
| Data are shown as n (%) or median (interquartile range). Clinically important intubation was defined as one that met at least one of the following criteria: loss of consciousness or impaired consciousness (Glasgow Coma Scale < 8), hypotension (systolic arterial blood pressure < 90 mmHg or mean arterial blood pressure < 65 mmHg), respiratory rate > 40 /min, hypoxia with SpO2 < 92% despite FiO2 1.0 or pH < 7.35 by blood gas analysis despite FiO2 1.0. Treatment failure was defined as either intubation or death within 7 days \*Calculated only for intubated patients | | |

## Slide 4
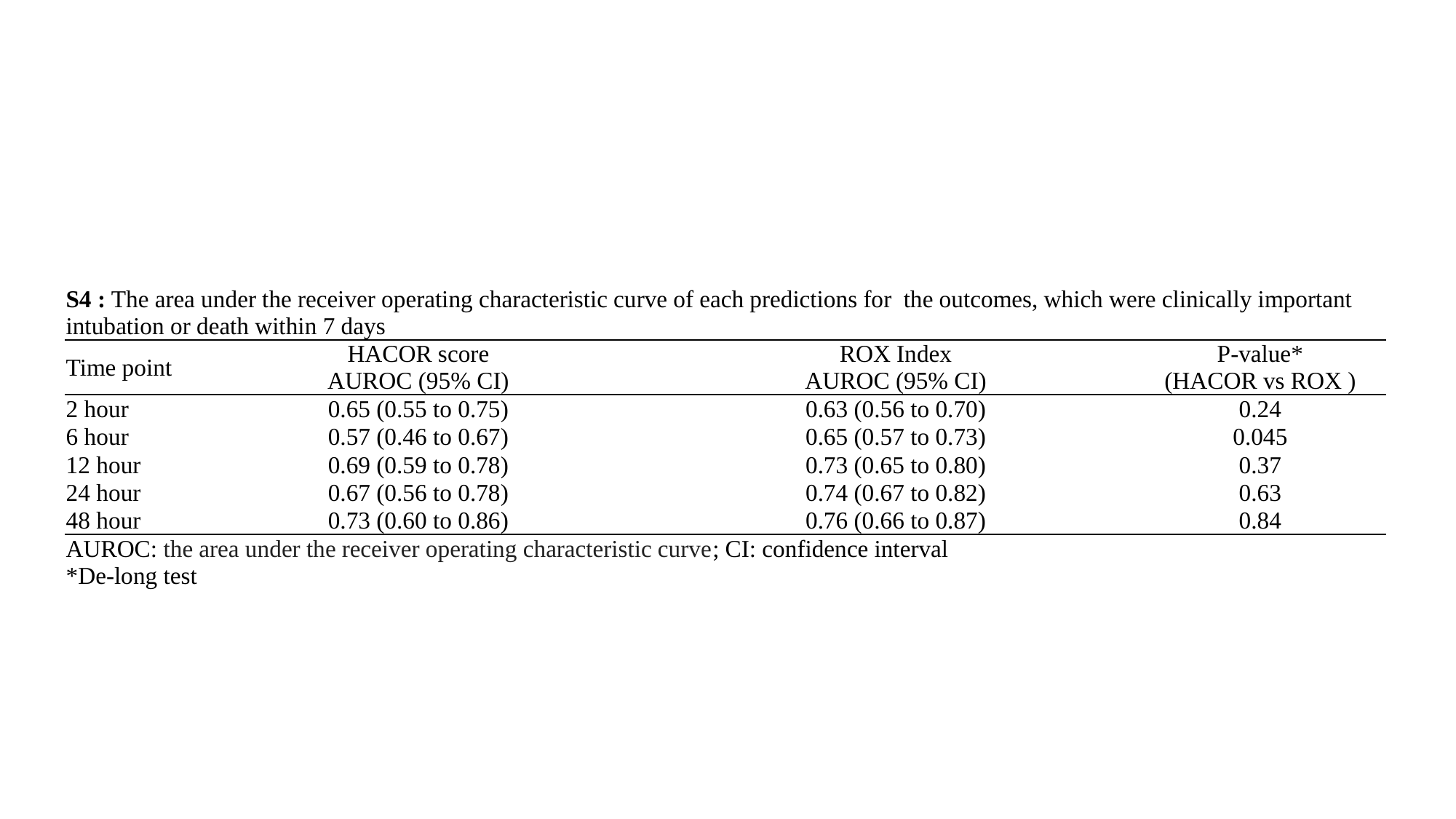

| S4 : The area under the receiver operating characteristic curve of each predictions for the outcomes, which were clinically important intubation or death within 7 days | | | |
| --- | --- | --- | --- |
| Time point | HACOR score AUROC (95% CI) | ROX Index AUROC (95% CI) | P-value\* (HACOR vs ROX ) |
| 2 hour | 0.65 (0.55 to 0.75) | 0.63 (0.56 to 0.70) | 0.24 |
| 6 hour | 0.57 (0.46 to 0.67) | 0.65 (0.57 to 0.73) | 0.045 |
| 12 hour | 0.69 (0.59 to 0.78) | 0.73 (0.65 to 0.80) | 0.37 |
| 24 hour | 0.67 (0.56 to 0.78) | 0.74 (0.67 to 0.82) | 0.63 |
| 48 hour | 0.73 (0.60 to 0.86) | 0.76 (0.66 to 0.87) | 0.84 |
| AUROC: the area under the receiver operating characteristic curve; CI: confidence interval \*De-long test | | | |

## Slide 5
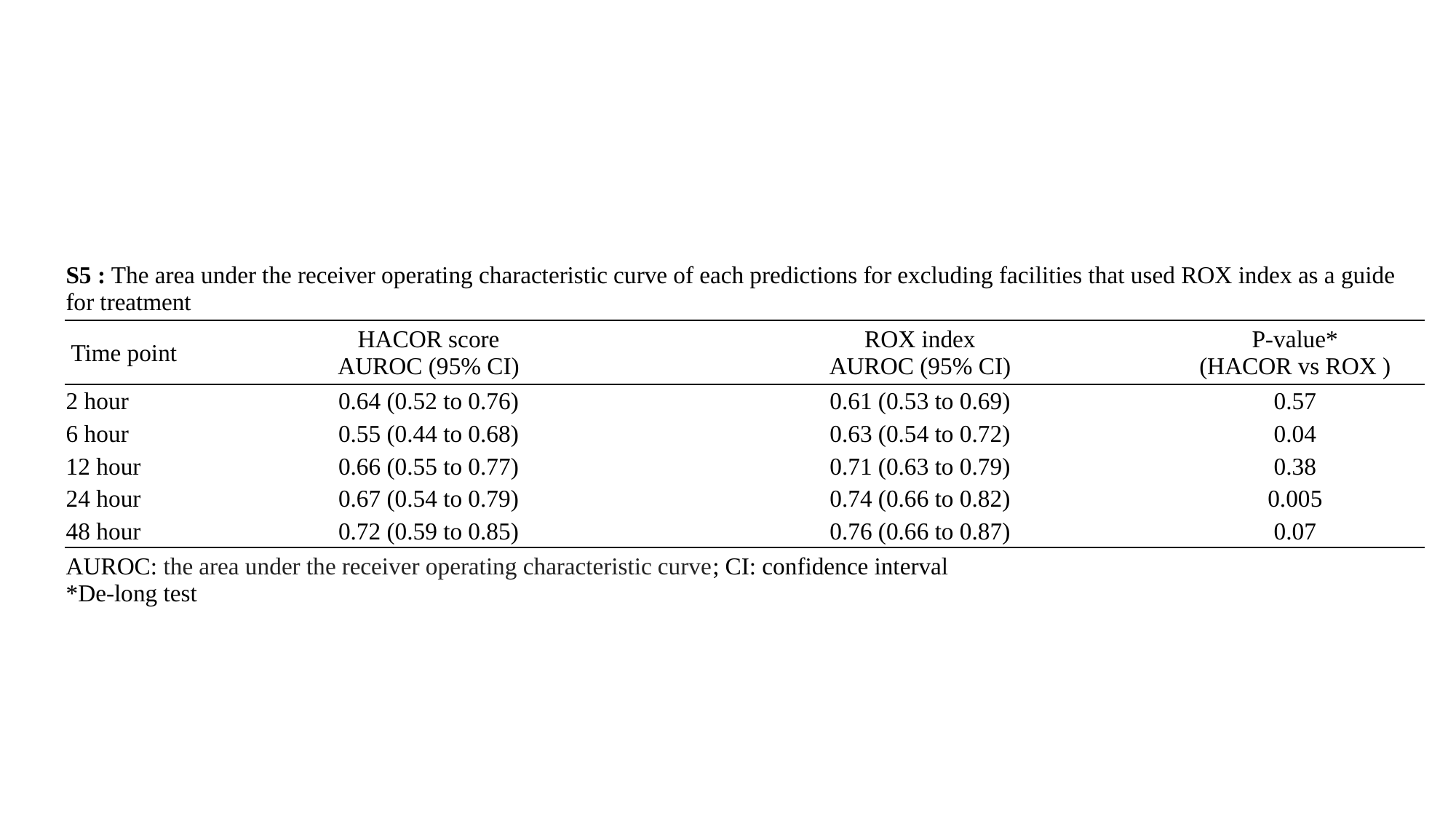

| S5 : The area under the receiver operating characteristic curve of each predictions for excluding facilities that used ROX index as a guide for treatment | | | |
| --- | --- | --- | --- |
| Time point | HACOR score AUROC (95% CI) | ROX index AUROC (95% CI) | P-value\* (HACOR vs ROX ) |
| 2 hour | 0.64 (0.52 to 0.76) | 0.61 (0.53 to 0.69) | 0.57 |
| 6 hour | 0.55 (0.44 to 0.68) | 0.63 (0.54 to 0.72) | 0.04 |
| 12 hour | 0.66 (0.55 to 0.77) | 0.71 (0.63 to 0.79) | 0.38 |
| 24 hour | 0.67 (0.54 to 0.79) | 0.74 (0.66 to 0.82) | 0.005 |
| 48 hour | 0.72 (0.59 to 0.85) | 0.76 (0.66 to 0.87) | 0.07 |
| AUROC: the area under the receiver operating characteristic curve; CI: confidence interval \*De-long test | | | |

## Slide 6
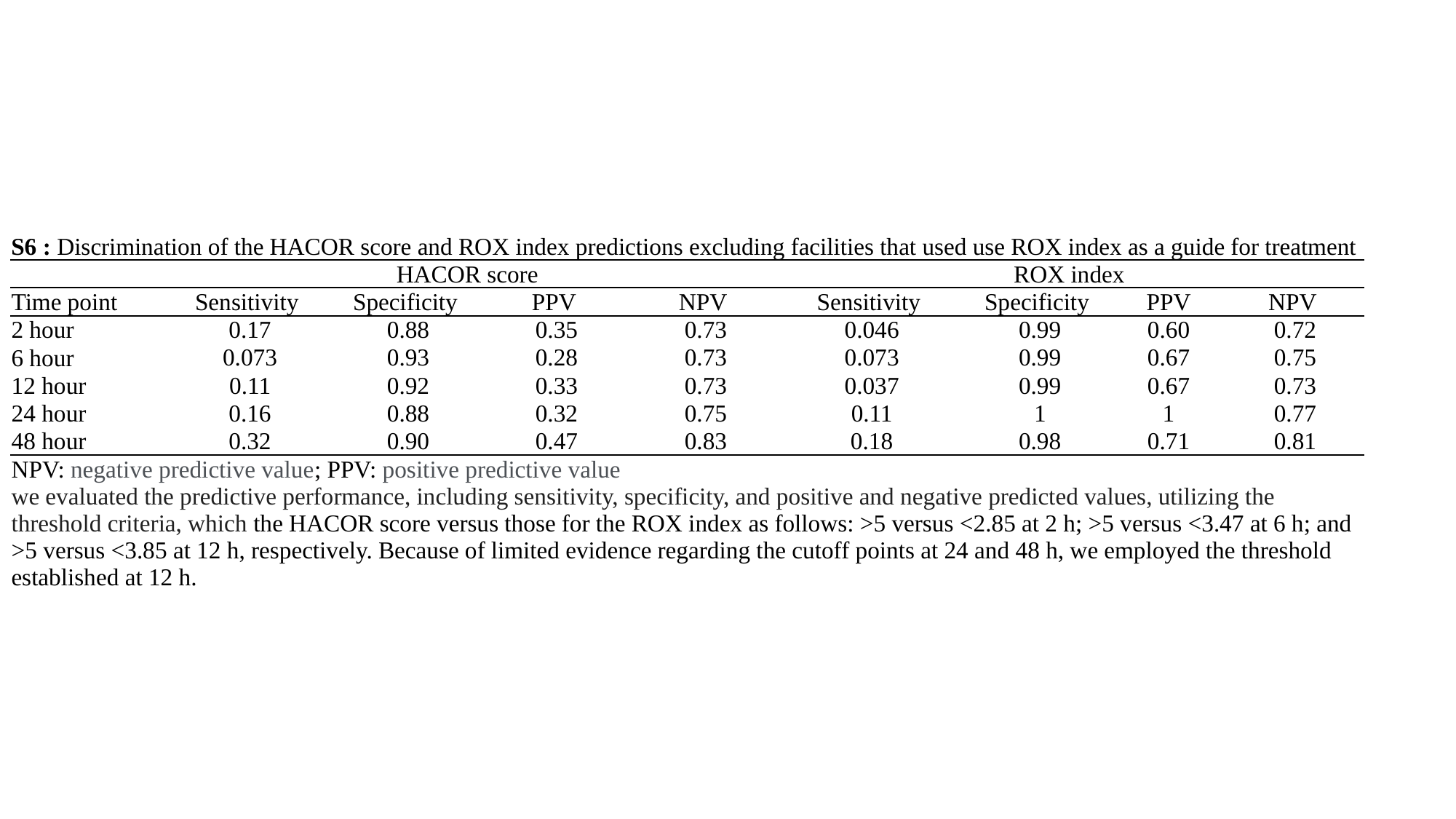

| S6 : Discrimination of the HACOR score and ROX index predictions excluding facilities that used use ROX index as a guide for treatment | | | | | | | | |
| --- | --- | --- | --- | --- | --- | --- | --- | --- |
| | HACOR score | | | | ROX index | | | |
| Time point | Sensitivity | Specificity | PPV | NPV | Sensitivity | Specificity | PPV | NPV |
| 2 hour | 0.17 | 0.88 | 0.35 | 0.73 | 0.046 | 0.99 | 0.60 | 0.72 |
| 6 hour | 0.073 | 0.93 | 0.28 | 0.73 | 0.073 | 0.99 | 0.67 | 0.75 |
| 12 hour | 0.11 | 0.92 | 0.33 | 0.73 | 0.037 | 0.99 | 0.67 | 0.73 |
| 24 hour | 0.16 | 0.88 | 0.32 | 0.75 | 0.11 | 1 | 1 | 0.77 |
| 48 hour | 0.32 | 0.90 | 0.47 | 0.83 | 0.18 | 0.98 | 0.71 | 0.81 |
| NPV: negative predictive value; PPV: positive predictive value we evaluated the predictive performance, including sensitivity, specificity, and positive and negative predicted values, utilizing the threshold criteria, which the HACOR score versus those for the ROX index as follows: >5 versus <2.85 at 2 h; >5 versus <3.47 at 6 h; and >5 versus <3.85 at 12 h, respectively. Because of limited evidence regarding the cutoff points at 24 and 48 h, we employed the threshold established at 12 h. | | | | | | | | |
